# Intelligent scaffolding for musical practice: a systematic review of AI and digital support for the self-regulated learning cycle

**DOI:** 10.3389/fpsyg.2026.1947824

**Published:** 2026-10-15

**Authors:** Xiuran Li, Mar Galera-Núñez, Yao Zhang

**Affiliations:** 1 School of Music and Dance, China West Normal University, Nanchong, China; 2 Faculty of Education Sciences, University of Seville, Seville, Spain

**Keywords:** artificial intelligence, intelligent scaffolding, metacognition, music education, self-regulated learning

## Abstract

**Introduction:**

Independent music practice depends on learners’ self-regulated learning (SRL), yet how digital tools and artificial intelligence (AI) support the forethought, performance, and self-reflection phases of the SRL cycle, and when such support may undermine learner autonomy, remains unclear.

**Methods:**

Following PRISMA 2020, we searched Web of Science, Scopus, ERIC, and PsycINFO through March 2026. Sixteen empirical studies met the inclusion criteria and were appraised with the Mixed Methods Appraisal Tool. Because of methodological heterogeneity, we conducted a thematic synthesis rather than a meta-analysis.

**Results:**

Three categories emerged: traditional digital tools, discriminative AI, and generative AI. Traditional tools supported planning and reflection; discriminative AI enabled real-time error detection; and generative AI may help learners manage task difficulty and translate expert feedback into actionable steps. Study quality was uneven, and several findings came from small-sample or cross-sectional designs, which limits causal inference. Two potential risks, over-scaffolding that may diminish autonomy and visual feedback that may divert attention from sound, were insufficiently studied.

**Discussion:**

Digital and AI tools appear to scaffold different phases of SRL, but learner autonomy should remain at the core. Fading explicit support and pedagogical guidance may help reduce dependence. Longitudinal studies are required to test these mechanisms.

## 1 Introduction

Traditional music education has for a long time operated through the master–apprentice model wherein students meet their teachers weekly for thirty to sixty minutes, yet according to researchers, effective skill development takes place in the home practice context (Brook and Upitis, 2015; Hanken, 2017; Boucher et al., 2021; Utermohl de Queiroz et al., 2024). Learners rarely achieve accurate execution on initial attempts. In the absence of the teacher, students have to follow directions, recognize their own mistakes and move on (Brook and Upitis, 2015; Acquilino and Scavone, 2022). Beginners and adolescents are especially prone to this phenomenon: lacking a strong inner aural image and the metacognitive ability to evaluate their performance, they fall into a pattern of mindless repetition, frustration, and eventual attrition (Wan et al., 2023; Li et al., 2025; Acquilino and Scavone, 2022). What ultimately determines whether solitary work is effective is the learner’s capacity for SRL—a theoretical framework developed by Zimmerman (2000), referring to the proactive processes by which individuals activate and sustain cognition, affect, and behavior in pursuit of personal goals (dos Santos Silva and Marinho, 2025). Rooted in social cognitive theory, SRL operates as a repeating cycle of forethought (goal-setting), performance (self-monitoring), and self-reflection, with metacognition as the internal engine throughout (Zimmerman, 2000; Ou et al., 2025; Preda-Uliță, 2025). This critical dependence on metacognition creates stark disparities in SRL proficiency: experienced musicians maintain high metacognitive awareness across the cycle, but novices rarely do (Wan et al., 2023; Li et al., 2025).

With the rapid development of Artificial Intelligence (AI) in recent years, researchers have found that AI can serve as a form of ‘intelligent scaffolding’ in the music practice environment: grounded in social constructivist theory, AI can deliver real-time, personalized feedback within a learner’s Zone of Proximal Development (ZPD) (Vygotsky, 1978; Peng et al., 2026), automating lower-level monitoring tasks such as pitch detection and rhythm tracking and thereby freeing attentional resources for higher-order concerns like expressive interpretation (Wood et al., 1976; Acquilino and Scavone, 2022). In theory, this sustains self-efficacy and autonomous practice habits (Li et al., 2025; Ou et al., 2025); in practice, the picture is more complicated. When learners hand over auditory monitoring entirely to AI, they face the risk of ‘automation complacency’—a gradual deterioration of internal vigilance that may hinder the internalization of core musical skills (Parasuraman and Manzey, 2010; Peng et al., 2026). Whether AI truly enhances autonomy or quietly breeds dependence remains an open question.

Several existing reviews have examined the role of technology in music education (e.g., Acquilino and Scavone, 2022; Merchán Sánchez-Jara et al., 2024), documenting a growing body of empirical research in this area. However, these reviews have not provided a systematic mapping of how different technologies correspond to the constituent stages of the self-regulated learning (SRL) process, leaving the psychological and functional mechanisms operating across the forethought–performance–self-reflection cycle insufficiently explored (Ou et al., 2025; Peng et al., 2026). Thus, the current challenge is not a lack of empirical studies, but rather a lack of integrative synthesis explaining how specific technological affordances support particular phases of SRL and when such scaffolding may shift toward learner dependence (dos Santos Silva and Marinho, 2025; Peng et al., 2026).

Peng et al. (2026) primarily examine the empowerment-dependence paradox from the perspective of learners’ self-beliefs and cognitive agency, focusing on intelligent assessment and generative artificial intelligence. In contrast, this review conducts a more detailed, staged analysis, dissecting how three distinct technology categories (traditional digital, discriminative AI, generative AI) intervene across each stage of Zimmerman’s (2000) forethought–performance–self-reflection cycle, and provides a structural explanation of the paradox rather than interpreting it at the level of psychological beliefs.

To address the identified gaps and provide a nuanced understanding of AI’s role in music practice, this systematic review is guided by the following three research questions:

- RQ1: What are the principal functional categories of digital tools and AI technologies currently applied to independent music practice and learning?
- RQ2: How do different categories of AI and digital tools (e.g., assessment-oriented, generative, and adaptive systems) specifically support the individual phases of the SRL cycle (Forethought, Performance, and Self-reflection) in musical practice?
- RQ3: How do these intelligent scaffolds influence learners’ psychological development (e.g., self-efficacy and metacognition), and under what conditions does this support lead to technological empowerment rather than cognitive dependency?

## 2 Theoretical framework

To understand how AI intersects with musical practice requires more than a single framework. Self-Regulated Learning draws together cognitive, metacognitive, behavioral, motivational, and emotional threads, and any account ignoring this complexity will inevitably be incomplete (Panadero, 2017; Antonini Philippe et al., 2020; Utermohl de Queiroz et al., 2024). Zimmerman’s cyclical model offers a structural map but cannot on its own explain what drives practice from within, or what happens when technology enters the picture (Alexander et al., 2011; Peng et al., 2026; Zimmerman and Tsikalas, 2005). Metacognition fills that gap: self-regulation is widely understood as the active, outward expression of metacognitive monitoring and control, functioning as the internal engine of the whole cycle (McPherson and Zimmerman, 2011; Brook and Upitis, 2015). Regarding how technology actually intervenes, this review draws on Vygotsky’s ZPD and the scaffolding literature (Wood et al., 1976; Vygotsky, 1978; Wang and Zhang, 2025). Concurrently, Cognitive Load Theory and cognitive offloading provide more precise mechanisms for understanding this shift. There is a shift in focus to expression and artistic evaluation when AI takes up the low-level monitoring (Sweller, 2011; Risko and Gilbert, 2016; Merchán Sánchez-Jara et al., 2024). As a result, both of these models assist us in identifying whether AI truly encourages independence in learners or it substitutes it, which could cause cognitive atrophy (Peng et al., 2026).

### 2.1 Zimmerman’s cyclical model of self-regulated learning

How does a musician learn to practice effectively in the absence of an in-person instructor? The essence of self-regulation lies in intent: the ability to think, feel and act in alignment with predefined goals, and to adapt in response to ongoing practice (Zimmerman, 2000; dos Santos Silva and Marinho, 2025). This review adopts Zimmerman’s cyclical model as its core organizing framework because it provides a systematic mechanism for translating internal psychological processes into observable behaviors (Alexander et al., 2011; Peng et al., 2026; Zimmerman, 1998). The cyclical nature of the model aligns with the iterative plan-execute-assess cycles inherent to instrumental practice (Varela et al., 2016; Jørgensen, 2004). Beyond this structural compatibility, Zimmerman’s framework is not restricted to purely cognitive and information-processing theory, instead, it centers on motivational beliefs – especially self-efficacy. This focus is uniquely valuable for explaining the motivational lulls and feelings of loneliness that frequently plague musicians during solo instrumental practice (McPherson and Zimmerman, 2011; Peng et al., 2026).

To fully understand how digital technology and AI systems operate within independent music creation practices, it is necessary to break down the three interrelated stages that constitute this closed loop, as well as the underlying detailed processes corresponding to each stage:

- Forethought phase: it occurs prior to practical hands-on exercises and sets the initial conditions for the learning activity. This stage is mainly divided into two categories: The first is task analysis, which includes formulating specific short-term learning objectives and planning learning strategies and methods (e.g., slowing down to study challenging music theory passages or using a metronome to assist practice); the second is the self-motivational beliefs, supported by self-efficacy, outcome expectations, and intrinsic interest in the musical work. In the context of autonomous music learning, if learners lack the relevant abilities in this stage, they cannot establish a clear internal auditory goal model before performance.
- Performance phase (volitional control): this stage encompasses the various processes involved in the actual performance of an instrument, aiming to optimize performance engagement and maintain focus. It includes self-control mechanisms (achieved through self-instruction, attention focus, and the application of task strategies) and self-observation mechanisms (specifically referring to real-time metacognitive monitoring and self-recording). In this stage, performers need to continuously devote precise auditory discrimination effort to promptly identify subtle deviations in intonation, rhythm, or timbre. The mode of simultaneous performance and monitoring generates a very high cognitive load, often exceeding the attentional capacity limits of novice musicians.
- Self-reflection phase: this mechanism is initiated immediately after task completion, retrospectively evaluating the results achieved to guide subsequent actions. It consists of two components: self-judgment and self-reaction. Self-judgment includes self-evaluation (comparing actual performance to established standards or ideal benchmarks) and causal attribution (determining the causes of success or failure); self-reaction refers to the emotional response of self-satisfaction as well as adaptive or defensive decision-making behaviors. If a student, following a technical error, makes a rigid and incorrect causal attribution (e.g., attributing the mistake to ‘insufficient innate talent’ rather than ‘ineffective practice strategies’), it can lead to frustration, subsequently weakening self-efficacy and disrupting the anticipatory phase of the next cycle.

The cyclical dimension of this model is precisely reflected in the fact that a performer’s self-feedback directly alters their motivation beliefs and practice goals for the next practice session. Therefore, a detailed analysis of these subprocesses is crucial for this study, providing a core analytical framework for exploring how traditional tools, discriminative AI, and generative AI inherit, externalize, or reinforce each of these cognitive stages.

### 2.2 Metacognition: the cognitive foundation of practice

As indicated by Flavell (1979), metacognition is the understanding of one’s own knowledge and the ability to regulate one’s cognitive processes accordingly. In music learning especially, the idea takes on a special significance. To improve, a musician must hear a problem in their own playing, name it with precision, and select a strategy that addresses the root issue instead of simply repeating the same passage (Preda-Uliță, 2025; Concina, 2019). Various studies demonstrate that proficient musicians tend to be powerful metacognitive monitors, and this consciousness has been shown to predict learning results far better than practice duration alone (Peynircioğlu et al., 2014; Li et al., 2025). Novices who lack developed audiation, rarely possess this awareness, mistakes go unheeded and make repetition pointless (Hallam et al., 2012; Wan et al., 2023; Bathgate et al., 2012). Therefore, well-organized feedback and thoughtful reflection are two key bases to foster development of metacognition competence and true musical autonomy (Li et al., 2025; Preda-Uliță, 2025).

### 2.3 Intelligent scaffolding and the zone of proximal development

Wood et al. (1976) introduced scaffolding to describe how a more capable partner absorbs the parts of a task a learner cannot yet manage. The theoretical grounding for this is proposed by Vygotsky’s (1978) ZPD, which represents the productive gap between what a learner can do independently and what becomes possible with support (Shabani et al., 2010; Wang and Zhang, 2025). AI can be seamlessly integrated into this theoretical framework. Personalized, real-time feedback enables learners to manage tasks that would otherwise be unmanageable for them independently. It translates vague auditory feedback from performance into precise, actionable signals, which support learners to set clear learning objectives, and in turn, develop their own internal performance standards (Peng et al., 2026). The defining feature of scaffolding in this AI-enabled context is that it is intentionally designed to be temporary, to ensure that the learner will ultimately no longer require external support (Sharma and Hannafin, 2007; Peng et al., 2026).

### 2.4 Cognitive offloading and the empowerment-dependency paradox

The concept of pushing computational work into tools to minimize the load on working memory is called cognitive offloading (Risko and Gilbert, 2016). In theory, this offers significant benefits for music learners. While AI takes over routine monitoring and control tasks, the learner can direct their focus toward articulation, expressive intent, and artistic musical decision-making (Sweller, 2011; Merchán Sánchez-Jara et al., 2024; Peng et al., 2026). Nevertheless, this shift carries inherent potential downsides. In cases where external feedback becomes the sole criterion a student uses to evaluate their performance, it may lead to the impairment of their internal auditory monitoring skills. ‘Automation complacency’—a gradual erosion of attentiveness from routinely trusting a system to do the noticing—could produce a musician who is technically proficient with AI assistance but entirely dependent on it (Parasuraman and Manzey, 2010; Peng et al., 2026). Therefore, developers must ensure that these AI tools serve as a bridge to learner autonomy, rather than a complete substitute for human judgment.

### 2.5 Synthesis of the theoretical roadmap

The four frameworks can be conceptualized as an interconnected hierarchical structure, in which each tier is grounded in the underlying framework that supports it (Peng et al., 2026). Zimmerman’s SRL framework provides the overarching structural blueprint, with metacognition serving as its core internal engine. When this core internal driver is not yet fully developed, Vygotsky’s ZPD and the associated theory of scaffolding delineate the mechanism through which targeted external support can be appropriately introduced. Finally, cognitive load theory outlines the specific conditions required for this technological support to reinforce internal regulation instead of overriding it (Flavell, 1979; Wood et al., 1976; Risko and Gilbert, 2016; Sweller, 2011). Figure 1 maps this nested relationship in detail. It should be emphasized that these four frameworks are applied throughout this review as interpretative lenses for organizing and making sense of the synthesis; the included studies were not designed to test Zimmerman’s model, ZPD/scaffolding, or cognitive load theory directly, and the frameworks themselves are not thereby empirically validated by this review. Where the Results and Discussion sections describe findings ‘in line with’ or ‘consistent with’ a given framework, this denotes conceptual fit rather than a confirmatory empirical test.

**Figure 1:**
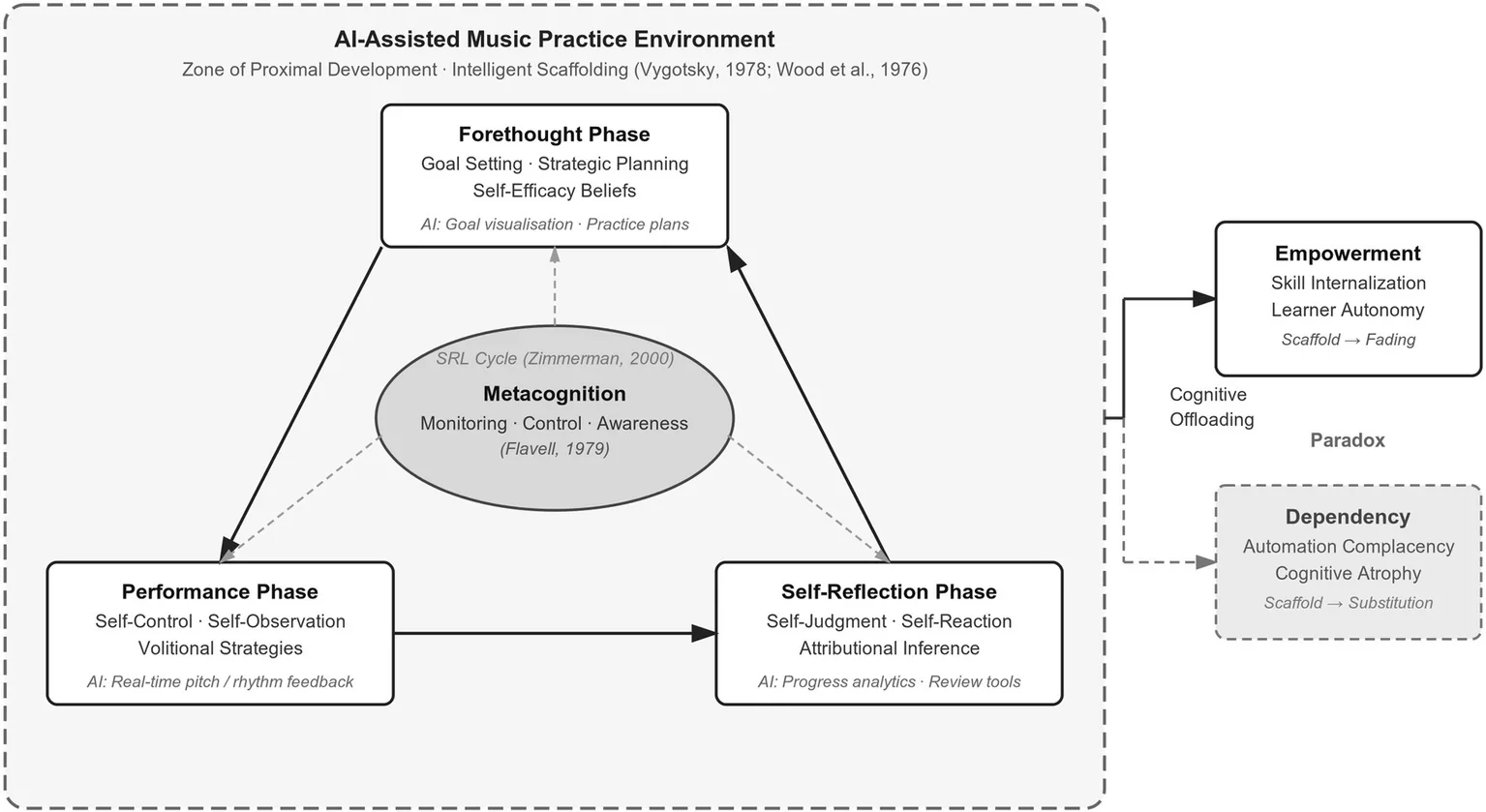
Integrated theoretical framework for AI-assisted musical self-regulated learning (Source: Authors’ own elaboration). Solid arrows indicate the primary SRL cycle; grey dashed arrows indicate metacognitive underpinning of each phase; the tinted box and dashed pathway indicate the dependency risk.

## 3 Methodology

This systematic review was conducted in accordance with the PRISMA 2020 statement (Page et al., 2021). Using a structured search, clear eligibility criteria, and systematic data extraction, we aimed to produce a full and reproducible account of how AI and digital tools have been used to scaffold SRL in music practice—with the requirement that included studies link a digital or AI intervention to at least one phase of Zimmerman’s SRL cycle and an associated metacognitive or motivational variable.

### 3.1 Search strategy

Four electronic databases were selected: Web of Science, Scopus, ERIC, and PsycINFO—chosen to span education, psychology, and music research. No publication date restriction was applied; searches ran from each database’s earliest records through March 2026, since foundational pre-AI work remains relevant to understanding how digital scaffolding has evolved. Search terms were grouped into three thematic blocks—Context, Intervention, and Outcomes—combined with Boolean logic. In line with PRISMA transparency requirements, the following string formed the basis of all database queries:

(‘music education’ OR ‘music practice’ OR ‘instrumental learning’ OR ‘piano practice’ OR ‘music learning’ OR ‘musical instrument*’) AND (‘artificial intelligence’ OR ‘AI’ OR ‘generative AI’ OR ‘large language model*’ OR ‘ChatGPT’ OR ‘digital tool*’ OR ‘educational technology’ OR ‘mobile app*’) AND (‘self-regulated learning’ OR ‘SRL’ OR ‘self-regulation’ OR ‘metacognition’ OR ‘self-reflection’ OR ‘self-monitoring’)

The string was adapted to each database’s syntax so that searches were confined to titles, abstracts, and keywords. Backward citation tracing across all included studies served as a supplementary snowballing measure.

### 3.2 Inclusion and exclusion criteria

The review included peer-reviewed empirical journal articles and full conference proceedings in English; conference abstracts, book chapters, editorials, preprints, and non-English publications were excluded. Eligibility criteria were developed through the PICOS framework (Population, Intervention, Comparison, Outcomes, Study Design). The full criteria are presented in Table 1.

**Table 1:** Inclusion and exclusion criteria based on the PICOS framework.

PICOS element	Inclusion criteria	Exclusion criteria
Population (P)	Music learners of all ages and proficiency levels, including young beginners, conservatoire students, undergraduate music majors, and pre-service music teachers.	Studies involving non-human subjects or learners outside the music domain were excluded.
Intervention (I)	Studies applying AI technologies (e.g., generative AI, discriminative real-time error-detection AI) or digital support tools (e.g., e-portfolios, video feedback, digital listening tools) within music practice or instruction contexts.	Studies limited to traditional face-to-face instruction without any digital technology component, or technology applications unrelated to music practice (e.g., AI-driven commercial composition tools), were excluded.
Comparison (C)	No strict comparator required. Studies could include control groups (traditional instruction), alternative technologies, or be single-group interventions/surveys.	N/A (Studies were not excluded based on the absence of a control group).
Outcomes (O)	Studies explicitly measuring or examining at least one construct within Zimmerman’s three-phase SRL model, including goal-setting, learning motivation, self-efficacy, real-time monitoring, emotional regulation, or self-evaluation.	Studies not addressing self-regulation or metacognitive variables were excluded.
Study Design (S)	Empirical studies with clearly reported data, encompassing quantitative designs (experiments, quasi-experiments, surveys) and qualitative designs (case studies, interviews, observations).	Purely theoretical papers, literature reviews, editorials, and system design specifications without human-subjects testing were excluded.

No strict requirements were imposed on control groups, since the goal was mapping AI tools across all SRL phases; included studies therefore span controlled designs, single-group interventions, surveys, and qualitative case investigations.

### 3.3 Study selection and PRISMA flow

The screening process adhered to the PRISMA 2020 flow (Figure 2). An initial database search returned 145 records; following deduplication, 18 records were removed, leaving 127 unique citations for title and abstract screening. Two researchers independently screened these 127 records using Rayyan (Ouzzani et al., 2016), excluding 109 outside scope and retrieving the remaining 18 in full text for detailed eligibility assessment. Inter-rater reliability was exceptionally high (Cohen’s *κ* = 0.82, 95% CI [0.66, 0.97]); minor discrepancies were resolved through discussion. Three articles were ultimately excluded—two for non-empirical design, one for a technological intervention unconnected to everyday musical practice. Backward citation searching identified one further eligible record: Boucher et al. (2021). In total, 16 empirical studies cleared every screening stage and proceeded to data extraction. Of these 16 studies, two were full conference proceedings (Feng et al., 2025; Xu and Fan, 2025); their eligibility as full peer-reviewed proceedings, rather than conference abstracts, was confirmed through reviewer consensus discussion prior to inclusion.

**Figure 2:**
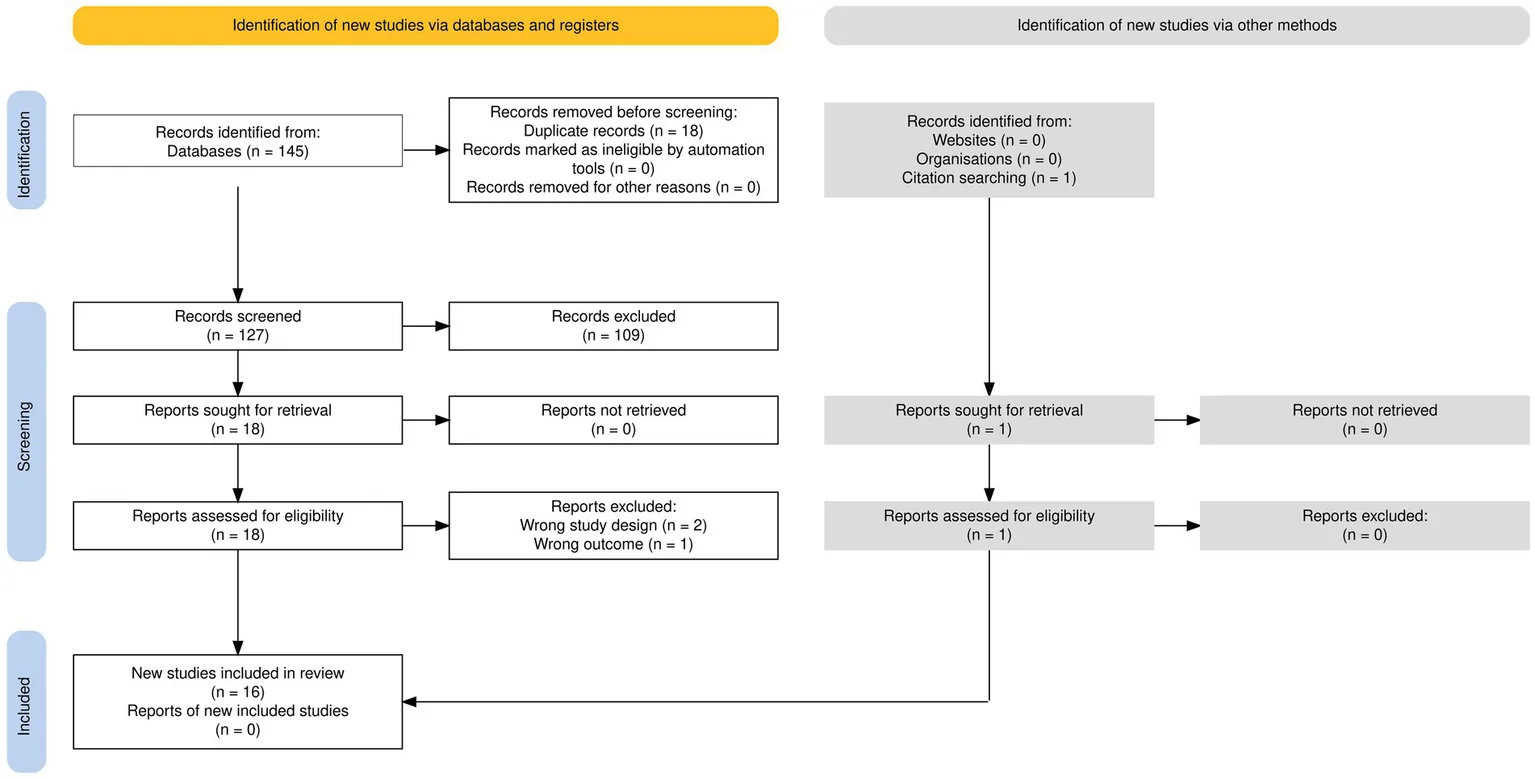
PRISMA 2020 flow diagram depicting the systematic search and selection process (*N* = 16 included studies).

### 3.4 Data extraction and analysis

For each of the 16 studies, data were recorded in a structured extraction matrix capturing study characteristics (authors, year, design), sample characteristics (N, participant profile, instrument/domain), technology profile (Generative AI, Discriminative AI, or Traditional Digital Tool), SRL theoretical mapping (Zimmerman phase(s) addressed and specific variables measured), and key findings including mechanisms of scaffolding.

This review employed thematic synthesis (Thomas and Harden, 2008) as the analytical framework, for three main reasons. First, the 16 studies included showed a lot of methodological diversity, with different designs from localized qualitative case studies (e.g., Wan et al., 2023) to large-scale cross-sectional surveys (e.g., Wang and Liu, 2025). This made a quantitative meta-analysis inappropriate and possibly misleading (Page et al., 2021; Thomas and Harden, 2008). Second, thematic synthesis aims to generate new interpretive insights from qualitative and mixed-methods data while preserving transparency and replicability—an approach widely validated in education and social sciences when the goal is to identify patterns, mechanisms, and contextual factors rather than pooled effect sizes (Thomas and Harden, 2008). Third, this method aligns directly with the review’s aim of mapping how different tool categories relate to the phases of Zimmerman’s SRL cycle, allowing the emergence of analytical themes (the three functional tool categories) directly from the data rather than imposing a pre-determined structure. The decision was further supported by previous systematic reviews in music education and technology-enhanced learning that successfully employed thematic synthesis under similar conditions of heterogeneity (e.g., dos Santos Silva and Marinho, 2025; Merchán Sánchez-Jara et al., 2024). It should be noted at the outset that a corpus of 16 studies, while adequate for thematic synthesis, represents a snapshot of a still-emerging research area rather than a fully representative sample of AI-assisted music practice research; the corpus is skewed toward studies published in English within the past three years (see Section 4.1.1) and toward specific instruments and learner populations (see Section 5.1), and readers should bear these representativeness constraints in mind when interpreting the thematic patterns reported below, rather than treating this caveat as confined to the Limitations section alone.

The analytic procedure for this review followed the three-stage model of thematic synthesis outlined by Thomas and Harden (2008), a method specifically designed to integrate findings from diverse study designs while maintaining transparency and analytical depth.

- Stage 1 (Line-by-line coding): Inductive codes were assigned line-by-line to the findings, discussion, and conclusion sections of all 16 included studies. These codes captured tool functions (e.g., ‘audio comparison tool’, ‘video playback’, ‘real-time pitch tracking’), learner outcomes (e.g., ‘improved singing performance’, ‘music theory acquisition’), and underlying psychological mechanisms (e.g., ‘self-efficacy maintenance’, ‘metacognitive awareness’, ‘flow induction’).
- Stage 2 (Developing descriptive themes): Through iterative comparison, initial codes were grouped into broader descriptive categories that identified common technological affordances across studies. For instance, codes relating to ‘note-by-note identification’, ‘gesture tracking’, and ‘automated scoring’ were synthesized under the descriptive theme of ‘Discriminative AI—real-time error detection’. Similarly, codes involving ‘personalized dialogue’ and ‘concept clarification’ were grouped under ‘Generative AI—strategic interaction’. This three-category typology (traditional digital tools, discriminative AI, generative AI) was therefore generated inductively from the coded data rather than imposed as an *a priori* taxonomy; it was not adopted from an existing classification scheme. Because several tools combine discriminative and generative components (e.g., hybrid error-detection systems with natural-language explanation layers), each study was classified according to its primary, most theoretically salient mechanism as reported by the study authors, and the category boundaries should be read as functional rather than strictly technical.
- Stage 3 (Generating analytical themes): In the final stage, descriptive themes were mapped onto Zimmerman’s (2000) three-phase SRL framework—forethought, performance, and self-reflection. This phase involved a ‘beyond-the-evidence’ synthesis to determine how specific technological affordances functioned as psychological scaffolds (e.g., mapping generative AI’s feedback to ‘strategy-translation scaffolds’ in the reflection phase). This stage produced the tool-by-phase matrix used to structure the Results section.

Two researchers independently reviewed the coding at each stage; discrepancies were resolved through discussion until consensus was reached. An audit trail documenting all coding decisions was maintained throughout the process to ensure transparency and reproducibility.

### 3.5 Quality assessment

All 16 studies were appraised independently by two researchers using the Mixed Methods Appraisal Tool (MMAT; Hong et al., 2018), selected for its capacity to accommodate five methodological categories under a shared evaluative framework. Initial inter-rater agreement was highly robust (87.5%, Cohen’s *κ* = 0.82); remaining disagreements were resolved through discussion. Quality across the corpus was uneven: four studies met all or nearly all criteria (Wan et al., 2023; Utermohl de Queiroz et al., 2024; Brook and Upitis, 2015; Li et al., 2025), while Xu and Fan (2025) received two No ratings and three Cannot tell owing to insufficient reporting of participant characteristics and outcome data. The most common shortcomings were inadequate control for confounding variables among non-RCT studies and limited sampling transparency in large-scale surveys. Readers should treat effect-size estimates and causal claims from affected studies with appropriate caution. Full ratings appear in Table 2.

**Table 2:** MMAT quality assessment summary table.

Author and year	Study category	S1	S2	C1	C2	C3	C4	C5
Li et al. (2025)	5. Mixed methods	✓	✓	✓	✓	✓	?	✓
Wang and Zhang (2025)	4. Quant. Descriptive	✓	✓	✓	?	✓	?	✓
Wang and Liu (2025)	4. Quant. Descriptive	✓	✓	?	?	✓	?	✓
Zhou and Kim (2024)	3. Quant. Non-RCT	✓	✓	✓	?	✓	*×*	✓
Ren et al. (2024)	3. Quant. Non-RCT	✓	✓	?	✓	✓	*×*	✓
Yuan (2024)	3. Quant. Non-RCT	✓	✓	?	✓	✓	*×*	✓
Ou et al. (2025)	5. Mixed Methods	✓	✓	✓	✓	✓	?	✓
Feng et al. (2025)	3. Quant. Non-RCT	✓	✓	?	✓	✓	✓	✓
Xu and Fan (2025)	4. Quant. Descriptive	✓	?	*×*	?	?	*×*	✓
Utermohl De Queiroz et al. (2024)	1. Qualitative	✓	✓	✓	✓	✓	✓	✓
Wan et al. (2023)	1. Qualitative	✓	✓	✓	✓	✓	✓	✓
Huang (2023)	3. Quant. Non-RCT	✓	✓	?	✓	✓	*×*	✓
Boucher et al. (2021)	2. Quant. RCT	✓	✓	*×*	✓	✓	?	✓
Brook and Upitis (2015)	5. Mixed Methods	✓	✓	✓	✓	✓	✓	✓
Portowitz et al. (2014)	5. Mixed Methods	✓	✓	?	?	✓	?	✓
Upitis et al. (2012)	1. Qualitative	✓	✓	✓	✓	✓	?	?

The table uses the following symbols: “*✓*” for yes, “*×*” for no, and “?” for cannot tell. MMAT Appraisal Criteria Legend.

Screening Questions (Applicable to all studies): S1: Are there clear research questions? S2: Do the collected data allow to address the research questions?

Category-specific Criteria (C1–C5): (1) Qualitative Studies: C1–Is the qualitative approach appropriate; C2–Are data collection methods adequate; C3–Are findings adequately derived from data; C4–Is there coherence between data, analysis and interpretation; C5–Is the researcher’s influence (reflexivity) addressed. (2) Quantitative RCTs: C1–Is randomization appropriately performed; C2–Are groups comparable at baseline; C3–Are outcome data complete; C4–Are assessors blinded (where applicable); C5–Did participants adhere to the assigned intervention. (3) Quantitative Non-RCTs: C1–Are participants representative of the target population; C2–Are measurements appropriate; C3–Are outcome data complete; C4–Are confounders accounted for; C5–Was the intervention administered as intended. (4) Quantitative Descriptive: C1–Is the sampling strategy relevant to the research question; C2–Is the sample representative; C3–Are measurements appropriate; C4–Is the risk of non-response bias low; C5–Is the statistical analysis appropriate. (5) Mixed Methods: C1–Is there an adequate rationale for using mixed methods; C2–Are the components effectively integrated; C3–Are the outputs of integration adequately interpreted; C4–Are divergences between qualitative and quantitative results addressed; C5–Do the different components adhere to quality criteria.

This review followed the PRISMA 2020 reporting guidelines; however, it was not prospectively registered, and no formal review protocol was prepared prior to conducting the study.

## 4 Results

Following the PRISMA screening process, a total of 16 empirical studies were ultimately selected for this analysis (see Table 3). The research findings will be presented in the following order: an overview of the basic characteristics of the studies, a classification of the types of AI and digital tools, and an analysis of how these tools support the construction of autonomous learning cycles in music practice.

**Table 3:** Descriptive characteristics and data extraction matrix of included studies.

No.	Author and year	Publication type	Study design	Sample size (N)	Target group / age	Instrument / domain	Technology type / descriptive theme (stage 2)	Tool name and function	Targeted SRL phase	Measured SRL variables	Key findings and scaffolding effects
1	Li et al. (2025)	Journal (Frontiers in Psychology)	Experimental; mixed-methods	*N* = 80 (EG = 38, CG = 42)	Pre-service teachers (undergraduates)	Vocal / Singing	Generative AI—strategic interaction + Traditional digital tools—reflective practice support	Yuanbao (Tencent GenAI chatbot): dialogue feedback; audio comparison tool for self-assessment; self-reflection journal writing	Forethought ✓ Performance ✓ Self-reflection (all 3 phases) ✓	Metacognitive ability; singing performance	AI-assisted training significantly improved EG metacognitive ability (*p* < 0.05); both groups improved singing performance, but no significant interaction effect; AI functioned as scaffold through personalized feedback and deep reflection opportunities.
2	Brook and Upitis (2015)	Journal (Music Education Research)	Qualitative case study	*N* = 9 teachers + students	Independent music studio students (various ages)	Multiple instruments (studio)	Traditional digital tools—reflective practice support (e-portfolio)	iSCORE web-based e-portfolio: supports goal-setting, inter-lesson feedback, recording sharing, and progress monitoring	Forethought ✓ (goal-setting) Self-reflection ✓	Goal-setting; self-regulatory behavior; teacher–student communication	iSCORE helped students articulate goals more clearly and facilitated inter-lesson teacher feedback; enhanced self-regulatory behavior; tool bridged in-class and out-of-class learning, supporting structured independent practice.
3	Wan et al. (2023)	Journal (J. Research in Music Education)	Qualitative multiple case study (interpretive)	*N* = 5 (1 teacher + 4 students)	Children (8–10 years)	Piano	Traditional digital tools—reflective practice support (digital aural app)	Software application (unnamed): accessible, customizable aural learning experiences; recording playback and autonomous listening practice support	Performance ✓ (self-monitoring) Self-reflection ✓	Self-regulation; aural perception; problem-solving; self-monitoring	Digital aural tools promoted autonomous out-of-class listening, enhanced aural familiarity and problem-solving; SRL development depended on individual learning preferences and continuous teacher guidance; tool scaffolded learning by providing accessible aural references.
4	Yuan (2024)	Journal (J. Computer Assisted Learning)	Experimental (pre-post test)	*N* = 98 (EG = 49, CG = 49; Chinese and Korean)	Undergraduates (18–20 years)	Piano / Polyphonic music composition	Generative AI—strategic interaction (deep learning model)	DeepBach graphical model: AI-assisted polyphonic composition; pseudo-Gibbs sampling; harmonic composition support	Forethought ✓ (learning motivation)	Academic motivation (learning interest, goal orientation)	EG mean motivation score (27.97) significantly higher than CG; AI composition tool enhanced motivation through creative engagement and modern technology integration; teachers reported improved theory–practice connection.
5	Feng et al. (2025)	Conference (HCII 2025, LNCS)	Quasi-experimental	*N* = 27 (aged 7–12, randomly assigned)	Children (7–12 years)	Music literacy (note reading)	Discriminative AI—real-time error detection (image recognition)	AI-guided music building block system: Roboflow + YOLOv11 deep learning image recognition (99.5% precision); interactive storybook + intelligent music blocks	Performance ✓ (engagement and emotion)	Learning engagement; achievement emotions (AEQ): enjoyment, hope, pride, boredom, shame, hopelessness	EG positive emotions (enjoyment, hope, pride) significantly higher than CG; negative emotions (boredom, shame, hopelessness) significantly reduced; AI system reduced music symbol cognitive barriers through immediate visual feedback, functioning as cognitive scaffold.
6	Wang and Zhang (2025)	Journal (Frontiers in Psychology)	Quantitative survey; SEM	*N* = 1,125 (9 institutions, music majors)	College students (18–22 years; UG/PG)	Music composition	Generative AI—strategic interaction (multiple tools)	MusicVAE, Transformer-based tools; Chinese university AI composition platforms; used for harmonic generation, melody composition, style exploration	Performance ✓ Self-reflection ✓	Music self-efficacy (MSE); music emotional intelligence (MEI); music creativity (MC)	AI use had significant positive direct effect on music creativity (β = 0.349, *p* = 0.001); MSE and MEI served as significant chain mediators; AI functioned as cognitive collaborator expanding ‘conceptual space’ rather than a mere tool.
7	Ou et al. (2025)	Journal (Frontiers in Psychology)	Quasi-experimental (4 months)	*N* = 40 (EG = 20, CG = 20; violin majors)	Conservatory violin students	Violin	Discriminative AI—real-time error detection (AI practice app)	AI-assisted practice app (unnamed): real-time pitch/rhythm feedback, practice records, adaptive practice suggestions	Forethought ✓ Performance ✓ Self-reflection ✓ (all 3 phases)	Music learning self-efficacy (MLSE); music performance self-efficacy (MPSE); performance outcomes; SRL three-phase strategies	AI enabled EG to maintain stable MLSE under increasing task difficulty; MPSE significantly improved (large effect size); EG performance significantly improved while CG declined; AI scaffolded all three SRL phases.
8	Ren et al. (2024)	Journal (J. Technical Education and Training)	Within-group experiment (pre-post test)	*N* = 44 (vocational; M22/F22; aged 14–17)	Vocational school students (14–17 years)	Music listening + sight-reading	Discriminative AI—real-time error detection + Traditional digital tools—reflective practice support	Kimi Assistant AI (music listening); Laiyinzhipu notation software (sight-reading); targeting resource-scarce disadvantaged areas	Performance ✓ (skill performance)	Music listening scores (melody, structure); sight-reading ability; learning attitude/motivation	AI significantly promoted only ‘melody’ and ‘structure’ in listening; all sight-reading indicators significantly improved; learning attitude significantly improved (*p* < 0.05); AI served as resource scaffold compensating for inadequate teaching conditions.
9	Portowitz et al. (2014)	Journal (Int’l Journal of Music Edu.)	Mixed methods (quasi-experimental intervention design)	*N* = 84 (Israel: EG = 40, CG = 22; US: *N* = 22)	Children (9–10 years, elementary)	Comprehensive music (understanding + composition)	Traditional digital tools—reflective practice support (educational software)	TLE (Teach, Learn, Evaluate!) software: music analysis, aural training; Impromptu software: digital composition and reflective writing	Performance ✓ (self-regulation)	Working memory; self-regulation; cognitive flexibility	In Harmony model successfully scaffolded music teaching; working memory, self-regulation, and cognitive flexibility all improved; software cultivated impulse inhibition and focused learning through structured task guidance.
10	Xu and Fan (2025)	Conference (DCSL 2024, LAIS 54)	Technology evaluation (system validation)	*N* = 10–100 (piano performance task samples); learner participant sample size not explicitly reported	College piano beginners	Piano	Discriminative AI—real-time error detection (CNN real-time error correction)	AI piano recognition and real-time error correction: 4 modules (audio acquisition/processing/recognition/display); CNN + MFCC feature extraction; instant error annotation and improvement suggestions	Performance ✓ (real-time feedback / self-correction)	Error recognition rate; learning efficiency; self-assessment ability; user satisfaction	Recognition speed and recall superior to traditional SVM; user satisfaction significantly higher; real-time feedback enabled precise error identification and correction, reducing error accumulation; AI as external error-correction scaffold.
11	Zhou and Kim (2024)	Journal (Education and Info. Technologies)	Experimental (random grouping)	*N* = 74 (Korean Chugye University undergraduates)	Undergraduate arts students	Music knowledge (comprehensive theory)	Generative AI—strategic interaction (LLM)	ChatGPT-4: conversational music theory explanation, personalized learning suggestions, performance technique guidance	Forethought ✓ (learning goals) Performance ✓ (knowledge acquisition)	Music knowledge mastery; learning attitude; learning satisfaction	EG music knowledge acquisition significantly superior to CG; ChatGPT-4 group showed significant pre-to-post improvement; students preferred learning with ChatGPT-4; tool scaffolded knowledge acquisition through personalized dialogue support.
12	Utermohl de Queiroz et al. (2024)	Journal (Research Studies in Music Edu.)	Qualitative case study (coding analysis)	*N* = 12 (5 teachers + 7 students)	String students (various developmental levels)	Strings (violin, etc.)	Traditional digital tools—reflective practice support (online teaching platform)	Online music lesson platform (Zoom, etc.): recording tasks, digital tool-assisted practice planning, repertoire discussion; no specialized SRL guidance tool	Forethought ✓ Performance ✓ (indirect support)	Motivation; self-efficacy; task strategies; metacognition	Teachers supported SRL only indirectly (recording tasks, repertoire discussion); lack of direct metacognitive guidance led to insufficient metacognitive development; gap in student-centered SRL instruction online; need to upgrade pedagogy toward explicit SRL instruction.
13	Upitis et al. (2012)	Journal (J. Instructional Pedagogies)	Mixed methods (3-phase development)	Ph.1: 1 T + 1S; Ph.2: 4 T + 15S; Ph.3: more users	Independent music studio students (various ages)	Multiple instruments (studio)	Traditional digital tools—reflective practice support (e-portfolio)	ePEARL e-portfolio (studio version): goal-setting, progress monitoring, recording sharing, reflection journal; later developed into iSCORE	Forethought ✓ (goals/planning) Performance ✓ Self-reflection ✓ (all 3 phases)	Goal-setting; self-monitoring; self-reflection; comprehensive SRL	ePEARL supported students in clarifying goals, connecting in-class and out-of-class learning; teachers provided out-of-class guidance via tool; tool scaffolded all three SRL phases, driving subsequent development of iSCORE.
14	Huang (2023)	Journal (Current Psychology)	Experimental (pre-post test)	*N* = 87 (M41/F46; aged 18–21)	University music students (18–21 years)	Score writing (electronic music composition)	Traditional digital tools—reflective practice support (notation software)	Overture notation software: score writing and reading, composition assistance, visualized music learning	Forethought ✓ (motivation/goals) Performance ✓	Learning motivation; attention; self-confidence; learning satisfaction	Overture significantly improved motivation (+14.3%), attention (+29%), self-confidence (+12.5%), satisfaction (+12.4%) (all *p* < 0.05); functioned as motivational scaffold through visual feedback and personalized learning modes.
15	Wang and Liu (2025)	Journal (European Journal of Education)	Quantitative survey; SEM; review + empirical	*N* = 941 (UG 49.7%; MA 36.2%; PhD 14.0%)	University music majors (UG/MA/PhD)	Comprehensive music (AI-assisted learning)	Generative AI—strategic interaction (Chinese GenAI platforms)	DeepSeek, Doubao (iFlytek), ERNIE Bot: generating musical ideas, analyzing composition, providing creative inspiration	Performance ✓ (engagement/flow) Self-reflection ✓ (efficiency perception)	Technophilia; positive experience; flow; learning efficiency perception	Both technophilia and positive experience significantly enhanced flow and learning efficiency; flow as key mediator (*p* < 0.001); positive experience→flow→learning efficiency path confirmed; AI as both emotional and cognitive scaffold by triggering flow state.
16	Boucher et al. (2021)	Journal (Psychology of Music)	Quantitative randomized controlled trial (RCT)	*N* = 16 (EG = 8, CG = 8; classical guitarists)	Intermediate–advanced university classical guitar students	Classical guitar	Traditional digital tools—reflective practice support (video recording playback)	Video recording feedback tool: 4 video recordings over 10 practice sessions; student self-assessment of performance recordings	Self-reflection ✓ (self-assessment / self-monitoring)	Self-assessment quality; self-monitoring behavior; self-evaluative comments	Video feedback significantly changed the way students expressed self-evaluative comments; more pronounced in advanced learners; video served as external monitoring scaffold, compensating for attentional limitations of the dual-task (execution + monitoring) during performance.

EG = Experimental Group; CG = Control Group; SRL = Self-Regulated Learning; AI = Artificial Intelligence; LLM = Large Language Model; CNN = Convolutional Neural Network; SEM = Structural Equation Modeling; UG = Undergraduate; PG = Postgraduate; MA = Master; T = Teacher; S = Student; n.d. = No date.

### 4.1 Descriptive characteristics of included studies

After conducting a cross-study analysis of the 16 included articles, it was found that this research field has developed rapidly in recent years, while also exhibiting significant diversity in terms of research methods and research contexts.

#### 4.1.1 Publication years and research trajectory

As shown in Figure 3, the studies included span the period from 2012 to 2025, yet their publication distribution shows a marked imbalance: over 70% of the literature was published between 2023 and 2025 (e.g., Li et al., 2025; Ou et al., 2025; Wang and Liu, 2025). This concentration of publications corresponds closely with the widespread adoption of generative AI tools and the rapid advancement of AI technologies. Consequently, AI-assisted music SRL has emerged as a frontier research focus at the intersection of educational technology and music psychology, rather than a subfield with a long-standing research foundation and mature development.

**Figure 3:**
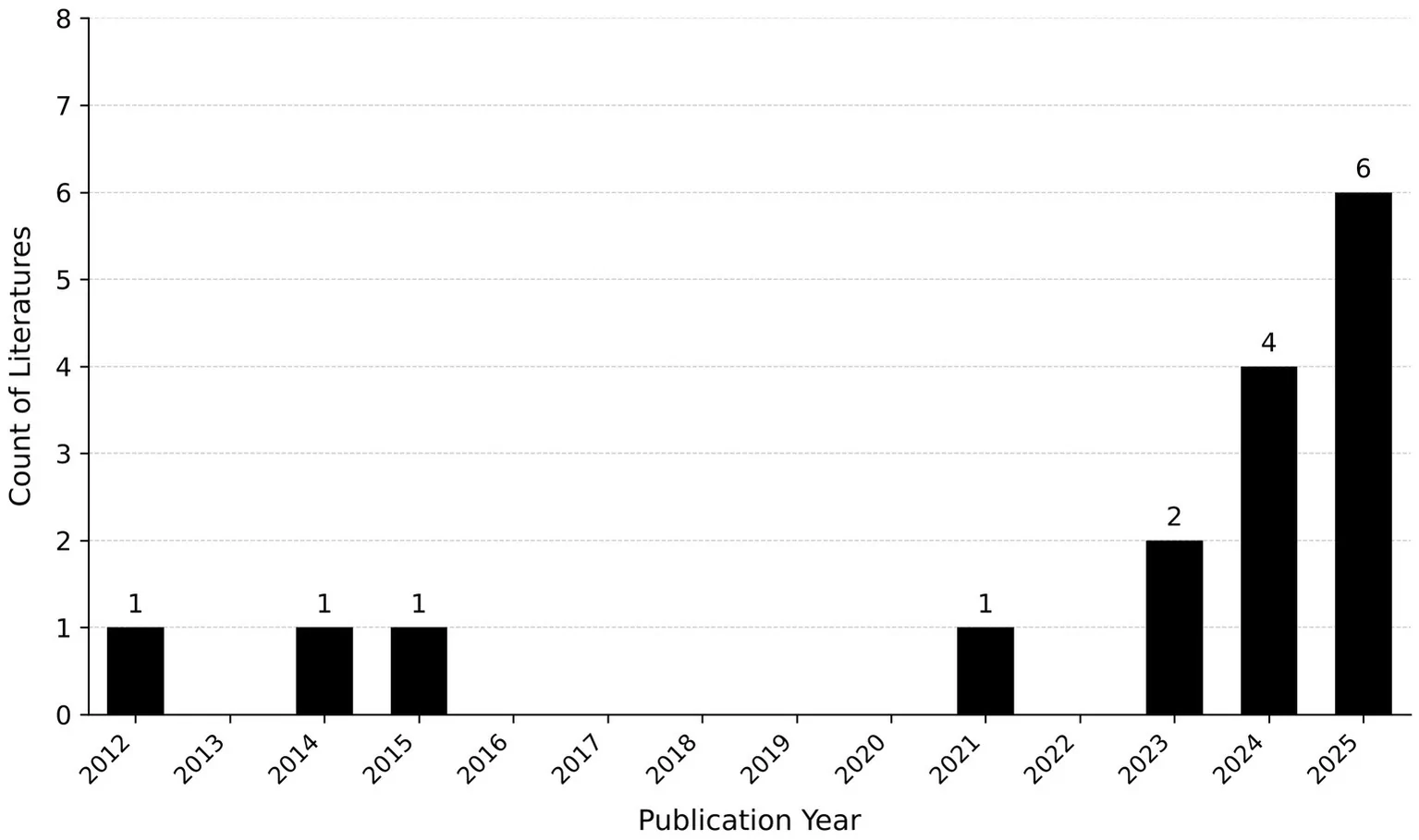
Distribution of included studies by publication year (2012–2025). *n* = 16 papers included in the systematic review. Based on data extraction results in Table 3.

#### 4.1.2 Participant profiles and sample design

The studies cover a wide range of developmental stages and professional levels. Research participants include children aged 7 to 12 (Feng et al., 2025; Wan et al., 2023), vocational school students (Ren et al., 2024), general university and conservatory students (Zhou and Kim, 2024; Wang and Liu, 2025; Huang, 2023), in-service professional development teachers (Li et al., 2025), as well as university guitar learners with performance experience (Boucher et al., 2021). The sample sizes of the studies also vary significantly: Wan et al. (2023) conducted a small-scale qualitative multiple-case study involving four students and their instructors, whereas Wang and Liu (2025) and Wang and Zhang (2025) conducted large-scale quantitative survey studies, employing structural equation modeling for statistical analysis of the survey data, with participant numbers reaching 941 and 1,125, respectively.

#### 4.1.3 Instruments and learning contexts

The scope of musical fields covered by these studies far exceeds expectations. The research by Wan et al. (2023) and Xu and Fan (2025) involves keyboard instruments; the studies by Ou et al. (2025) and Utermohl de Queiroz et al. (2024) focus on string instruments; Li et al. (2025) concentrate on vocal training; Boucher et al. (2021) conduct research on the guitar; while the studies by Feng et al. (2025), Ren et al. (2024), Huang (2023), and Yuan (2024) cover music theory, music appreciation, and digital composition. The diverse musical research contexts indicate that the scaffolding functions examined are not unique to any single instrument, but rather reflect a general mechanism by which intelligent tools play a role in the autonomous regulation process of music skill learning.

### 4.2 Typology of AI and digital scaffolds

The interventions across the 16 studies differ in underlying cognitive and pedagogical logic—specifically in how each tool manages learners’ cognitive load and positions itself within the SRL cycle (Ren et al., 2024). Drawing on algorithmic architecture, mode of interaction, and feedback type, three functional categories can be distinguished, though it is worth noting that some tools contain elements of more than one. A concrete example illustrates the distinction: a discriminative AI system, such as a real-time pitch-tracking tool, listens to a learner’s performance and classifies each note as in-tune or out-of-tune against a fixed reference—it discriminates between correct and incorrect outputs but does not generate new musical content. A generative AI system, such as an LLM-based practice assistant, instead composes a slowed-down accompaniment track for a difficult passage or produces a natural-language explanation of why a phrase sounds unresolved—it creates new content tailored to the learner’s situation rather than classifying an existing performance. Some tools blur this boundary (e.g., systems that pair real-time error detection with a generative explanatory layer), which is why category assignment in this review reflects each study’s primary, most theoretically salient mechanism (see Section 3.4). Figure 4 illustrates the distribution across the 16 studies.

**Figure 4:**
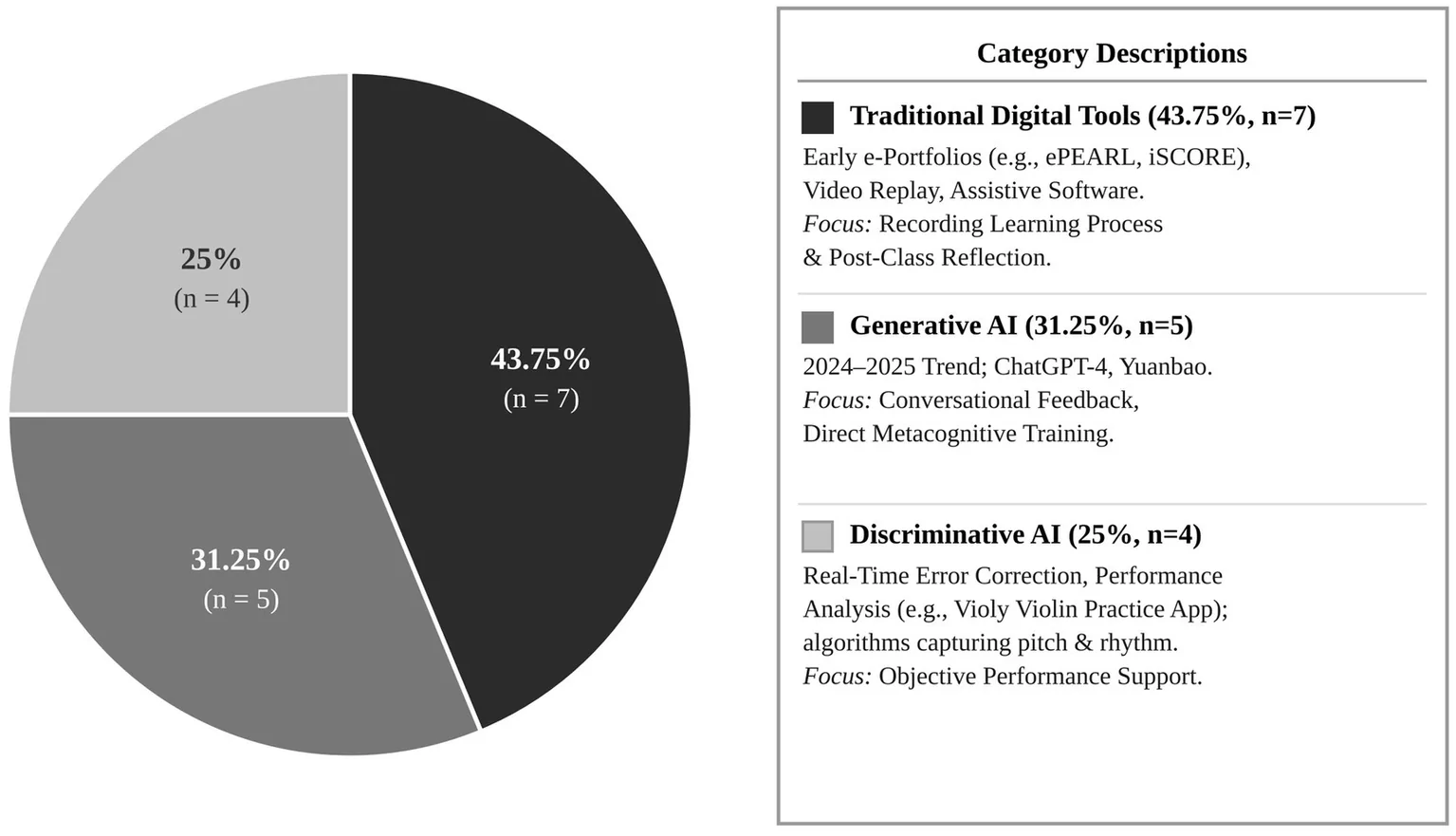
Distribution of digital and AI tools across included studies. *n* = 16 papers included in the systematic review. Based on data extraction results in Table 3.

#### 4.2.1 Traditional digital tools—recording and reflection scaffolds

These web-based or audio-visual platforms scaffold practice by creating an external record of what would otherwise remain tacit and inaccessible. Representative examples include *ePEARL* and *iSCORE* e-portfolios (Upitis et al., 2012; Brook and Upitis, 2015), the Sono digital listening tool (Wan et al., 2023), video feedback (Boucher et al., 2021), and Overture notation software (Huang, 2023). Their primary contribution is cognitive externalization—drawing out hidden intentions and performance data into a form the learner can actually inspect (Li et al., 2025; Ou et al., 2025). From a Zimmermanian perspective, this directly supports the forethought and self-reflection phases by converting fleeting mental states into durable, inspectable artefacts (Zimmerman, 2002; Hatfield et al., 2017; Flavell, 1979). E-portfolios prompt written, revisable goals (Brook and Upitis, 2015; Upitis et al., 2012); video recordings break the perceptual bottleneck of live performance, letting students observe technique with something closer to the objectivity of an outside eye (Boucher et al., 2021); digital listening platforms allow comparative self-assessment against professional recordings (Wan et al., 2023). The limitation recurring across this category is engagement. Without sustained internal motivation or teacher prompting, students often simply ignore the reflective affordances these tools provide (Utermohl de Queiroz et al., 2024; Brook and Upitis, 2015).

#### 4.2.2 Discriminative AI—real-time error detection and cognitive scaffolds

Tools in this category rely on deep learning models for audio signal processing or computer vision, classifying incoming performance data against a stored reference and flagging deviations at a speed no human listener could match. Representative systems include the *Violy* smart practice app (Ou et al., 2025), a YOLOv11-based AI building-block system (Feng et al., 2025), and a piano real-time error-correction platform (Xu and Fan, 2025). Ren et al. (2024) combined the Kimi assistant for music listening with Laiyinzhipu notation software for music reading. Although Kimi is built on a large language model, in this study it was used to analyze students’ listening responses and provide correct/incorrect feedback; in line with the primary-mechanism rule (Section 3.4), the study is therefore classified as discriminative AI combined with a traditional digital tool. Within the performance phase, these systems may function as protective scaffolds (Zimmerman, 2002; Ou et al., 2025): novice players frequently cannot hear their own errors at speed, and error-laden repetition tends to entrench rather than correct faulty motor patterns (Li et al., 2025; Acquilino and Scavone, 2022). By intercepting mistakes in the moment, discriminative AI avoids the social discomfort that in-person teacher correction often carries. In doing so, it creates what can be described as a psychologically safe, low-emotional-risk practice environment: a space in which students can make repeated errors without fear of being judged, penalized with poor evaluations, or exposed in front of peers—thereby protecting self-efficacy and motivation that might otherwise erode under socially evaluative pressure. From a cognitive load perspective, externalizing monitoring demands reallocates attentional capacity toward expressive and interpretive goals that would otherwise remain beyond reach (Sweller, 2011; Preda-Uliță, 2025; Wang and Zhang, 2025), precisely the mechanism Vygotsky’s ZPD anticipates (Shabani et al., 2010; Wan et al., 2023). Gains in self-efficacy reported by Ou et al. (2025) [Music Performance Self-Efficacy (MPSE) *p* < 0.017] are consistent with this protective function, though the causal pathway remains inferential.

#### 4.2.3 Generative AI—personalized interaction and motivational scaffolds

Generative AI tools—built on large language models (LLM) or generative adversarial networks (GAN)—represent a qualitatively different kind of scaffolding. Rather than detecting errors in a known performance space, they generate new content and engage learners in open dialogue. Representative tools include the *Yuanbao* AI chatbot (Li et al., 2025), *ChatGPT-4* (Zhou and Kim, 2024), the *DeepBach* polyphonic generation model (Yuan, 2024), and broader AI music creation platforms (Wang and Zhang, 2025; Wang and Liu, 2025). Where discriminative AI supports practice by watching and correcting, generative AI supports it by talking and creating alongside the learner (Wang and Zhang, 2025; Yuan, 2024). From a Self-Determination Theory standpoint, these tools intervene at the motivational core of the forethought phase: by lowering the perceived difficulty of compositional and interpretive tasks, they provide early access to competence experiences that satisfy the psychological need for efficacy (Evans, 2015; Wang and Liu, 2025; Bandura, 1997). For instance, when a beginning student encounters a complex harmonic progression or a polyphonic piece, a generative AI system can instantly produce a slowed-down accompaniment track, or an LLM-based assistant can explain, measure by measure, which specific technique to apply. By making the task more accessible, the student avoids immediate frustration and instead gains early access to competence experiences. As a virtual tutor, generative AI translates abstract, metaphor-laden expert feedback into concrete, actionable practice steps—documented by Li et al. (2025) in vocal training. As a co-creator, it removes technical barriers to compositional tasks such as multi-voice writing (Yuan, 2024); In this vein, Wang and Liu (2025) found that students engaged more deeply and absorbingly with AI tools when they perceived the technology as genuinely facilitating comprehension and improving their learning—a psychological phenomenon of total absorption known as “*flow*” (Csikszentmihalyi, 1990), and their cross-sectional SEM results are consistent with flow states acting as a mediator between AI engagement and learning efficiency, though the correlational design cannot rule out alternative directions of influence. The motivational scaffolding is thus indirect but systematic: by making difficult musical tasks tractable, generative AI helps maintain the positive affect and self-efficacy that sustain the SRL cycle across sessions (Wang and Zhang, 2025; Ou et al., 2025).

### 4.3 Impact of AI on SRL phases

SRL is a dynamic and iterative process that includes three phases: forethought, performance, and self-reflection (Zimmerman, 2000). The following will elaborate on the ways in which various tools can be applied in conjunction with these three phases.

#### 4.3.1 Forethought phase: goal-setting and the reconstruction of motivational beliefs

The forethought phase includes task analysis, goal setting, and the activation of self-motivational beliefs. In this initial stage, traditional digital tools can play a supportive role, helping learners to clearly and systematically organize their learning goals. In this regard, research by Brook and Upitis (2015) and Upitis et al. (2012) indicates that e-portfolios can transform students’ generally vague and abstract willingness to practice into written, visualized, and verifiable learning objectives. In a similar vein, research by Wan et al. (2023) found that the Sono Listening Tool helps child keyboard learners build a clear internal auditory cognitive model before actually performing a musical passage.

At the same time, generative AI systems can have a profound impact on this phase, effectively enhancing learners’ self-motivational beliefs and alleviating feelings of frustration. Wang and Zhang (2025) point out that such music generation technologies allow learners to engage with authentic and complex compositional outcomes in advance, which, under traditional teaching methods, would typically require years of professional technical accumulation. Related research by Yuan (2024) further supports this view, showing that AI-assisted multi-part composition technology significantly reduces the perceived difficulty of multi-part composition tasks and markedly increases learners’ learning motivation. Additionally, Huang (2023) confirmed that using score-assistive software such as overture can improve music students’ learning confidence by 12.5%.

Finally, discriminative AI also plays a key role in pre-class learning preparation. Studies by Ou et al. (2025) and Ren et al. (2024) suggest that such systems can help students set practice goals based on objective data, analyzing past error records, rather than planning blindly based on transient emotional states. This algorithm-based planning approach is associated with fewer of the repeated failures in learning that could otherwise undermine students’ sense of self-efficacy in music learning before they even start practicing independently (MPSE, *p* < 0.017).

#### 4.3.2 Performance phase: real-time monitoring, cognitive load, and flow

The performance phase is the stage within the SRL cycle where technological interventions are most concentrated and the evidence in empirical literature is most robust. A common conclusion in this field of study is that novice learners typically lack the metacognitive abilities required to independently identify their own practical errors. In this regard, research by Ou et al. (2025) found that the pitch-by-pitch tracking function applied by *Violy* can help learners detect performance deviations that are imperceptible to beginners. Additionally, the study by Feng et al. (2025) demonstrated that multisensory feedback provided by the YOLOv11-based system can reduce the cognitive load associated with self-monitoring, enhance learners’ enjoyment of performance, and simultaneously lower performance anxiety. Research by Xu and Fan (2025) confirmed that during the cultivation of learners’ foundational skills, technological interventions can effectively shorten the plateau phase, the period in which learners continue practicing while their progress temporarily stalls; however, Xu and Fan (2025) had several MMAT criteria rated “No” or “Cannot tell,” including an unreported learner sample size (Table 2; Section 3.5), so this finding is treated here as a sensitivity note rather than as evidence carrying equal weight to the other, more rigorously reported studies in this synthesis.

In terms of engagement during practice, the cross-sectional survey by Wang and Liu (2025) found that technophilia and positive experience with AI tools were positively associated with learners entering the optimal experience state (flow state) proposed by Csikszentmihalyi (1990), a psychological state of complete focus and immersion in learning activities; as with other SEM-based findings in this corpus, this association does not establish that AI use causes flow to occur. Based on this adaptive learning concept, research by Zhou and Kim (2024) found that, compared with the control group, personalized conceptual explanations provided by *ChatGPT-4* can significantly improve learners’ music theory exam scores (*p* = 0.000). Moreover, research by Li et al. (2025) discovered that the continuous, iterative multi-round question-and-answer interaction mode of the *Yuanbao* chatbot multi-turn dialogue can help vocal learners break down abstract practice goals into concrete practical actions step by step; research by Ren et al. (2024) found that AI-supported instruction in a vocational-school setting significantly improved only the melody and structure indicators of music listening, while all sight-reading indicators improved.

#### 4.3.3 Self-reflection phase: accurate self-evaluation and strategy reconstruction

The self-reflection phase (including self-assessment and feedback on one’s own performance) determines whether learners will adjust their learning strategies in the next practice session and the specific ways in which they do so (Zimmerman, 2000). In home-based autonomous learning contexts, this stage has long been the weakest part of the self-regulated learning process due to the lack of immediate teacher guidance. To address this issue, video feedback can directly break through learners’ cognitive bottlenecks. In this regard, Boucher et al. (2021) found that college guitar learners who watched recordings of their own performances were more willing and able to independently identify and correct performance errors. Moreover, Utermohl de Queiroz et al. (2024) indicated that video playback enables learners to step outside their own performance perspective and examine their performance from an external observer’s viewpoint, thereby uncovering technical habits and posture issues that were previously difficult to detect.

As for electronic learning portfolios, they extend this externalized presentation and dynamic tracking capability to a long-term dimension (Brook and Upitis, 2015; Wan et al., 2023). These platforms accumulate various forms of evidence of learning practices over time (such as audio recordings, past learning objectives, reflective journals, etc.), allowing learners to review their overall growth and development, rather than judging themselves solely based on isolated results from a single learning session. This cumulative record can effectively avoid the ‘recency effect,’ preventing learners from assessing their abilities solely based on a day of frustration or heightened emotions, thereby enabling accurate self-reflection. At the same time, this recording system provides a foundation for phased strategy optimization, allowing music learners to objectively identify medium to long term effective practice methods, discard inefficient learning habits, and readjust future learning goals based on solid practical experience.

At higher levels of automation, discriminative AI shifts the central focus of self-reflection from subjective experience to data-driven evaluation, representing an innovation in self-reflective practices. Research by Ou et al. (2025) confirms that the ‘Audition Report’ of the *Violy* can provide quantifiable feedback on pitch and rhythm accuracy. The system relies entirely on algorithms for assessment, thereby avoiding learners’ self-enhancement bias (the unconscious tendency to overestimate one’s performance to protect self-esteem) and eliminating the common politeness bias (teachers or peers, out of empathy or concern for others’ feelings, may dilute, embellish, or omit constructive criticism, leading to distorted feedback) present in human feedback.

Generative AI adds dimensions of interpretation and adaptation to this process. Li et al. (2025) found that the *Yuanbao* chatbot can reconstruct abstract singing instruction provided by teachers into concrete, step-by-step practical tasks, significantly enhancing students’ metacognitive levels (*p* = 0.03). Consistent with this conclusion, Zhou and Kim (2024) demonstrated that personalized explanations provided in natural language by *ChatGPT-4* allow students to maintain a calm and composed emotional state during reflection. Compared with traditional teaching methods, students’ mastery of theoretical knowledge showed a statistically significant improvement. The scaffolding teaching mechanisms corresponding to the aforementioned technologies are detailed in the comprehensive summary in Table 4.

**Table 4:** Mapping of intelligent scaffolding mechanisms across Zimmerman’s (2000) SRL cycle by tool category.

Tool category	Forethought phase	Performance phase	Self-reflection phase
Traditional digital tools	**Planning and goal setting** Using e-portfolios to articulate goals and ‘Listen mode’ to build aural models (Upitis et al., 2012; Brook and Upitis, 2015; Huang, 2023; Wan et al., 2023)	**Self-monitoring scaffolds** Objective self-observation via video playback and mouse-movement tracking (Upitis et al., 2012; Boucher et al., 2021; Utermohl de Queiroz et al., 2024)	**Perceptual scaffolds** Adopting a ‘third-person perspective’ via video/audio feedback to detect previously invisible habits (Boucher et al., 2021; Wan et al., 2023; Utermohl de Queiroz et al., 2024)
Discriminative AI	**Protective and planning scaffolds** Calibrating goals via historical data and buffering self-efficacy (MPSE) to prevent early practice frustration (Ren et al., 2024; Ou et al., 2025)	**Corrective scaffolds** Real-time pitch/rhythm tracking, visual error markers, and multi-sensory recognition (Ou et al., 2025; Xu and Fan, 2025; Feng et al., 2025)	**Diagnostic and evaluation scaffolds** Using data-driven reports to identify ‘hidden issues’ and provide an objective basis for goal recalibration (Ou et al., 2025; Ren et al., 2024; Xu and Fan, 2025)
Generative AI	**Motivational scaffolds** Lowering task complexity via auto-generation and boosting attention/confidence (Wang and Zhang, 2025; Yuan, 2024)	**Interactive strategy scaffolds** Personalized dialogic feedback and conceptual explanations to induce ‘flow’ and reduce cognitive load (Zhou and Kim, 2024; Wang and Liu, 2025; Li et al., 2025)	**Strategy-translation scaffolds** Reframing abstract instruction into concrete, sequenced action steps to enhance metacognitive awareness (Zhou and Kim, 2024; Li et al., 2025)

Bold text indicates the primary scaffolding function per phase. Based on thematic synthesis of 16 studies (*n* = 16).

## 5 Discussion and conclusions

The thematic synthesis shows that the progression is not smooth but coherent: digital and AI tools have moved from being peripheral supports to structurally significant supports in all three phases of Zimmerman’s (2000) SRL cycle. The newly-emerging tool types cannot be substituted. More specifically, traditional digital tools act primarily as scaffolds for planning and reflection, whereas discriminative AI can act as a kind of real-time, protective monitor during the performance phase. Generative AI then works as a scaffold that translates motivation and strategic planning into action while linking forethought and self-reflection. Study quality, as appraised per individual MMAT criterion in Table 2—consistent with Hong et al.’s (2018) guidance against collapsing MMAT results into a single composite score—was also uneven across the corpus. As detailed in Section 3.5, several studies met all or nearly all criteria, but inadequate control for confounding variables among non-RCT designs and limited sampling transparency in large-scale surveys were recurring weaknesses, and one study, Xu and Fan (2025), had multiple criteria rated “No” or “Cannot tell” because of insufficiently reported participant and outcome data. The synthesis below should be read with this variation in mind: findings drawn from studies with fewer unmet or unclear criteria carry more weight than those resting predominantly on Xu and Fan (2025) or on other studies with several ‘No’ or ‘Cannot tell’ ratings. The corpus provides reasonably consistent evidence, from quasi-experimental and experimental studies, that these tools are associated with short-term gains on the outcomes each study measured; the cross-sectional survey studies in the corpus (Wang and Zhang, 2025; Wang and Liu, 2025) further show that these gains correlate with self-efficacy and flow, but, given their designs, cannot establish that AI use causes these outcomes. In the discussion that follows we therefore distinguish, wherever possible, between findings directly supported by the included studies’ data, mechanisms proposed by the theoretical frameworks in Section 2, and plausible but as yet untested explanations, rather than treating all three as equally established. With this distinction in mind, the more pressing question is not whether these tools function, but what kinds of learning they deliver and at what cost.

### 5.1 Heterogeneity and transferability of findings

Before turning to specific interpretations, it is worth stating plainly that the 16 included studies differ from one another on nearly every dimension relevant to synthesis, and this heterogeneity should temper how the patterns below are read. The studies span markedly different musical domains and instructional contexts: piano (Wan et al., 2023; Xu and Fan, 2025), violin and other strings (Ou et al., 2025; Utermohl de Queiroz et al., 2024), voice (Li et al., 2025), guitar (Boucher et al., 2021), and general music theory, appreciation, or composition (Feng et al., 2025; Ren et al., 2024; Huang, 2023; Yuan, 2024), alongside several studies spanning multiple instruments or general AI-composition use (Brook and Upitis, 2015; Upitis et al., 2012; Wang and Liu, 2025; Wang and Zhang, 2025). Participant characteristics are similarly wide-ranging, from children as young as 7–8 years old (Feng et al., 2025; Wan et al., 2023) to adolescents in vocational programs (Ren et al., 2024) to university, conservatory, and in-service teacher populations, with sample sizes spanning two orders of magnitude, from a four-student qualitative case study (Wan et al., 2023) to cross-sectional surveys of 941 and 1,125 respondents (Wang and Liu, 2025; Wang and Zhang, 2025). Intervention duration and intensity vary just as widely, from single-session drawing or reflection tasks and a 10-session practice diary (Boucher et al., 2021) to a four-month, 32-session quasi-experimental program (Ou et al., 2025) to cross-sectional surveys that impose no intervention at all. Finally, the outcome measures themselves differ in kind rather than merely in instrument: some studies report validated psychometric scales of self-efficacy or flow (Ou et al., 2025; Wang and Liu, 2025), others report standardized cognitive-skill tests such as working memory and cognitive flexibility (Portowitz et al., 2014), others report exam scores or performance-error rates (Zhou and Kim, 2024; Xu and Fan, 2025), and others report qualitative codes derived from reflective logs or interviews (Wan et al., 2023; Utermohl de Queiroz et al., 2024).

This degree of heterogeneity means that the three functional tool categories and their mapping onto Zimmerman’s (2000) SRL cycle (Table 4) represent a thematic synthesis across fundamentally different methodological traditions, not a convergent effect replicated under comparable conditions. Where the Discussion below describes a pattern as recurring across studies, this should be read as recurring across this heterogeneous set of designs, populations, and outcome measures, rather than as a single effect independently confirmed multiple times. Findings drawn from studies that are closer in domain, population, or design to a reader’s own context of interest should be weighted more heavily than the aggregate pattern; the aggregate pattern is best understood as a map of where the field currently stands, rather than as an estimate of a single underlying effect size.

### 5.2 Psychological impact and conditions for empowerment in AI-assisted music practice

AI-based scaffolds mainly promote learners’ psychological development by enhancing self-efficacy and metacognitive awareness (Li et al., 2025; Peng et al., 2026). Music learning self-efficacy tends to decrease with the increase of repertoire difficulty under traditional conditions, while AI-assisted learners are more likely to maintain stable confidence and achieve significant improvement in MPSE—which Ou et al. (2025) attribute to the “objective mirror” of automated feedback. Musical self-efficacy also appears to play a mediating role: in a cross-sectional structural equation model, stronger ability beliefs were associated with more frequent AI use and, in turn, with higher musical creativity scores (Wang and Zhang, 2025); given the cross-sectional survey design, this mediation pattern should be read as a statistical association rather than a demonstrated causal chain. However, the shift from support to true empowerment is subject to the empowerment–dependency paradox (Peng et al., 2026). Several enabling conditions emerge from the corpus: teacher facilitation to transcode algorithmic data into actionable musical goals (Wan et al., 2023); active internalization of AI feedback through reflective journaling to prevent automation complacency (Li et al., 2025; Parasuraman and Manzey, 2010); satisfaction of psychological needs for competence and autonomy (Wang and Liu, 2025; Evans, 2015); and positioning AI as a supplementary ‘digital proxy’ that overcomes resource constraints without displacing learner agency or human guidance (Li et al., 2025; UNESCO, 2023; Yuan, 2024). It should be said that these conditions remain theoretically grounded hypotheses rather than fully tested experimental findings—a distinction future longitudinal research should address (Peng et al., 2026).

### 5.3 Theoretical implications: from static support to dynamic intelligent scaffolding

Wood et al. (1976) introduced scaffolding, likening it to a temporary, interpersonal support mechanism in the process of learners solving problems. The findings of this review greatly expand the connotation of this metaphor. Early digital tools in the research corpus, including electronic learning portfolios, recording devices, and notation software, fully align with the original concept of scaffolding instruction, providing asynchronous and static support frameworks for learning. Discriminative AI, as detailed in Section 4.2.2, brings a more fundamental transformation: by externalizing real-time monitoring functions that novice learners cannot yet perform themselves, it enacts what Wood et al. (1976) termed “marking critical features,” moving scaffolding support from the periphery of practice into the moment-to-moment activity itself. Generative AI represents a further qualitative step: as illustrated in Section 4.2.3, it enacts Wood et al.’s (1976) “task and tutee dependent” mode of scaffolding by translating abstract instructional language into concrete action through dialogue with the individual learner. Whether this dialogic mode constitutes genuine metacognitive regulation or a high-precision simulation of it remains to be rigorously investigated in future research.

### 5.4 Critical reflections: dependency, auditory atrophy, and the risk of over-scaffolding

#### 5.4.1 The displacement of auditory attention by visual feedback

Music is primarily an auditory art. The ability to construct clear auditory images in one’s mind —that is, hearing musical phrases in the mind before performing them— is widely regarded as the foundation for proficient independent musical practice (Wan et al., 2023). However, discriminative AI tools mainly convey information through visual channels, including colored indicators, on-screen scores, and dynamic visualizations of pitch deviations. For beginners, whose attention is already very limited, flashing red marks often capture their attention more instantly than the sounds produced by their fingertips. In the long term, this can lead to a risk of cognitive narrowing: learners may become increasingly adept at responding to visual cues, but habits of focused listening and the associated ability for internal self-monitoring may fail to develop. Notably, among the 16 experiments included in this research, none directly explored this substitution effect. This phenomenon itself is of significant research value: the academic community has long focused on the capabilities enhanced by AI but rarely on the various skills it may inadvertently displace or weaken. This study, based on the theory of attention resources (Sweller, 2011), presents this phenomenon as an observational finding and uses it to propose research hypotheses. Future studies should conduct targeted longitudinal research to track the dynamic changes over time in learners’ use of visual feedback and their auditory self-monitoring abilities.

#### 5.4.2 Over-scaffolding and the absence of fading mechanisms

The scaffolding model inherently carries a temporary core characteristic: the instructional support is designed to be gradually withdrawn as students’ capabilities improve, thereby transferring the responsibility for regulating learning progressively to the learners themselves (Wood et al., 1976). However, among all AI music application cases analyzed in this study, almost none adhere to this core design principle. The review of the current research found that, of the 16 tools included in the analysis, none implemented a fading mechanism for technology support. Ou et al. (2025) and Ren et al. (2024) confirmed that AI assistance can significantly enhance students’ learning performance while using the tools, but neither study examined learners’ learning behavior and self-directed capability development after the AI support was completely removed.

The absence of a fading mechanism in digital scaffolding can result in over-scaffolding, which, in turn, constrains the development of students’ artistic literacy. Tong et al. (2026) pointed out that AI systems can precisely correct objective, quantifiable musical parameters (such as accurate pitch and standard tempo), but aesthetic judgment and emotional expression remain domains of human experience. Addressing this limitation, Li et al. (2025) suggested that the feedback output by AI often represents merely the ‘average model’ or statistically optimal result derived from algorithm training data. Applied to musical practice, this means the software evaluates students’ performance using uniform, standardized corrective criteria. This teaching model presents notable pedagogical drawbacks and easily fosters surface-level learning behaviors in students: they mechanistically mimic and conform to the software’s data standards to avoid error prompts, rather than genuinely internalizing knowledge and improve their abilities. This hinders students from developing independent musical aesthetic judgment, forming self-directed interpretive thinking in performance, and shaping a unique personal artistic style, fundamentally deviating from the true essence of music learning.

### 5.5 Practical implications

The recommendations below are drawn from a corpus of 16 studies that is heterogeneous in design and, for several tools, still limited to a single small-sample or cross-sectional evaluation. They should therefore be read as provisional, evidence-informed starting points for practice and design rather than as firmly established prescriptions, pending confirmation from larger, higher-quality, and longer-duration intervention studies.

#### 5.5.1 For music educators: reframing the teacher’s role in a human-AI pedagogical partnership

The evidence does not support the conclusion that AI tools are substitutes for expert teaching. What it does support is the conclusion that they change what expert teaching needs to do. When a discriminative AI system handles real-time error detection during home practice, the lesson can shift toward interpretive, expressive, and motivational dimensions that no algorithm addresses well. When students arrive having practiced with generative AI assistance, the teacher’s task includes helping them interrogate and contextualize the feedback received, and build the critical literacy needed to use these tools purposefully rather than passively (Preda-Uliță, 2025). As Utermohl de Queiroz et al. (2024) note, there is an urgent need for instrumental teachers to receive formal training in SRL principles and the effective use of digital tools to foster students’ self-regulation in musical learning.

#### 5.5.2 For software developers: embedding SRL theory and fading mechanisms into the design architecture

The current generation of AI music applications has demonstrated that automated feedback can be accurate, positively reinforcing, and efficient. The next design challenge is to ensure that this feedback is grounded in pedagogical theory. Specifically, this requires incorporating a fading mechanism into the system’s logic: gradually reducing the frequency of feedback as competence increases, or periodically scheduling practice sessions without assistance. One concrete implementation plan is to introduce a ‘blind practice mode,’ in which visual feedback is disabled in approximately 20% of practice sessions, requiring users to conduct self-assessment before revealing the scores provided by the AI (Wood et al., 1976; Sharma and Hannafin, 2007). Wang and Zhang (2025) proposed that when designing the architecture of generative AI products, they should integrate users’ psychological growth trajectories, allowing the complexity of the tools to dynamically adapt as users build confidence. Equally important is emphasizing auditory design—creating tools that not only exercise users’ operational skills but also train their auditory perception, rather than merely using sound signals as a substitute for visual screen signals (Wan et al., 2023). Upitis et al. (2012) noted that scaffolded tools designed around a complete self-regulated learning cycle are more conducive to cultivating users’ self-regulation abilities—abilities that persist even as the underlying technology is updated or replaced.

### 5.6 Limitations and future directions

There are several important limitations concerning the interpretation of the study findings. To begin with, the 16 included studies are sufficient for thematic synthesis but not for strong generalization; the corpus is best viewed as a conceptual mapping of a nascent field rather than a settled account of established effects. As discussed in Section 5.1 (Heterogeneity and Transferability of Findings), the 16 included studies differ substantially in musical domain, participant characteristics, intervention duration, and outcome measures, and the number of randomized controlled trials remains limited. This heterogeneity constrains how far any single finding can be generalized beyond the specific context it comes from. The conclusions of this review are therefore most applicable to structured music-learning contexts that share characteristics with the included studies, particularly those involving technology-mediated feedback, guided practice, and learner reflection.

The review’s protocol was not prospectively registered on *PROSPERO* or *OSF*, which limits independent verification of the priori analytic plan. Grey literature (preprints, unpublished dissertations, and industry reports) was also not searched. Therefore, unpublished null or negative findings may be underrepresented in this synthesis, particularly given that the rapidly developing generative-AI literature may be vulnerable to publication and positive-result bias. Relatedly, most newer studies involving generative AI are either small-sample quasi-experimental designs or qualitative investigations that do not allow causal inference. More importantly, none was designed to test whether scaffolding gains persist after AI assistance is removed; therefore, this review maps how AI may support practice but cannot confirm whether such support develops durable independent musicianship. In addition, almost all SRL constructs were measured through learner questionnaires, which capture perceptions and beliefs about learning rather than actual practice behavior. Future research should incorporate multimodal behavioral indicators, such as eye-tracking and interaction logs, to complement self-report measures.

Another limitation of the synthesis is that it has been restricted almost exclusively to English-language publications. This language restriction means that research involving localized AI platforms and published in Chinese, Korean, Spanish, or other languages may have been excluded from the present synthesis. This limitation also introduces a temporal lag because AI tools are developing considerably faster than the peer-review cycle. Therefore, future syntheses may need to incorporate grey literature and computational approaches to better capture rapidly evolving developments in the field.

A related concern is that generative AI models themselves iterate far faster than the studies evaluating them. Several systems discussed in this review (e.g., *ChatGPT-4*, *DeepBach*) may be superseded, retrained, or discontinued within a few years, and newer models may exhibit substantially different feedback quality, reliability, or failure modes than those examined here. Consequently, tool-level findings synthesized in Section 4 should be treated as illustrative of a particular technological moment rather than as durable claims about specific products. What is more likely to remain stable is the phase-level functional logic identified in Table 4—the types of scaffolding functions (error detection, dialogic translation of feedback, and motivational task simplification) that discriminative and generative systems may provide in principle, regardless of which specific model implements them. Future reviews will need to revisit this mapping as new generations of AI tools emerge.

Taken together, the priorities raised throughout this review fall into three clusters. Methodologically, future work should prospectively register protocols, search grey literature, and adopt designs—ideally longitudinal studies or investigations incorporating deliberate fading manipulations—capable of testing whether scaffolding gains persist once AI support is withdrawn. Such studies should also incorporate multimodal behavioral data (e.g., eye-tracking and interaction logs) rather than relying exclusively on self-report questionnaires. Theoretically, subsequent studies should directly test, rather than merely illustrate, whether AI-mediated interaction constitutes genuine metacognitive regulation as opposed to a high-precision simulation of it (Section 5.3), and should examine the boundary conditions under which cognitive offloading shifts from empowerment to dependency (Section 2.4). Practically, priorities include developing and evaluating fading mechanisms and blind-practice modes (Section 5.5.2), as well as preparing music educators to interpret and contextualize AI-generated feedback for students (Section 5.5.1).

Overall, the 16 reviewed studies show that AI and digital tools have increasingly been examined not only as useful add-on features but also as potential structural supports across different phases of SRL. Systems focusing on assessment may support learner confidence through objective feedback, while generative AI tools may enable learners to engage in higher-order decisions concerning musical expression and creative intent. The empowerment–dependency paradox (Peng et al., 2026) remains central to understanding whether such scaffolding ultimately supports learner autonomy or inadvertently encourages dependence. When AI is embedded within a deliberately structured practice routine, it may create opportunities for engagement and the internalization of metacognitive strategies. Nonetheless, visual feedback’s potential interference with auditory attention and the widespread absence of fading mechanisms remain unresolved challenges. Further research should examine long-term learning trajectories and cross-cultural engagement to develop analytics capable of distinguishing metacognitive growth from simple compliance with AI guidance. When educators use AI carefully while maintaining essential human guidance, these technologies may better support the development of musicians who are technically competent, analytically engaged, and increasingly self-regulated (Preda-Uliță, 2025; Wang and Zhang, 2025; Peng et al., 2026).
