# Supplementary material for: Intelligent scaffolding for musical practice: a systematic review of AI and digital support for the self-regulated learning cycle
Source: Front Psychol. 2026 Oct 15;17:1947824. doi: 10.3389/fpsyg.2026.1947824 (PMC13717558; doi:10.3389/fpsyg.2026.1947824)
Supplement: Supplementary file 1 [file Data_Sheet_1.PDF]

PRISMA 2020 Checklist

Manuscript: "Intelligent Scaffolding for Musical Practice: A Systematic Review of AI and Digital Support for the Self-Regulated Learning Cycle"

Checklist adapted from Page et al. (2021), The PRISMA 2020 statement: An updated guideline for reporting systematic reviews. BMJ, 372, n71. https://doi.org/10.1136/bmj.n71

'Reported' column key: Yes = fully reported; Partial = addressed narratively but not as a formally pre-specified method; N/A = not applicable to this review.

Item #	Checklist item	Location in manuscript	Reported
**TITLE**			
1	Identify the report as a systematic review.	Title page — "A Systematic Review of AI and Digital Support for the Self-Regulated Learning Cycle"	Yes
**ABSTRACT**			
2	Provide a structured summary covering background, objectives, eligibility criteria, information sources, risk-of-bias assessment, synthesis methods, results, and main limitations.	Abstract, p. 1	Yes
**INTRODUCTION**			
3	Describe the rationale in the context of existing knowledge.	Section 1 (Introduction), paragraphs 1–3	Yes
4	Provide an explicit statement of objectives or questions the review addresses.	Section 1, RQ1–RQ3	Yes
**METHODS**			
5	Specify inclusion and exclusion criteria, and how studies were grouped for synthesis.	Section 3.2; Table 1 (PICOS framework)	Yes
6	List databases/registers searched, dates of coverage, and other sources consulted.	Section 3.1 (Web of Science, Scopus, ERIC, PsycINFO; searched through March 2026; backward citation searching)	Yes
7	Present the full search strategy for at least one database.	Section 3.1 (Boolean search string reported in full)	Yes
8	Describe the screening process, number and independence of reviewers, and any automation tools.	Section 3.3 (two independent reviewers, Rayyan, Cohen's $\kappa = 0.82$)	Yes
9	Describe methods for extracting data, number/independence of reviewers, and processes for obtaining data from study authors.	Section 3.4 (structured extraction matrix, two independent reviewers)	Yes
10a	List and define the outcomes for which data were sought.	Section 3.4 (SRL-phase mapping and measured SRL variables; Table 3)	Yes
10b	List and define other variables for which data were sought.	Section 3.4 (study, sample, and technology characteristics; Table 3)	Yes
11	Describe methods for assessing risk of bias, number/independence of assessors, and tools used.	Section 3.5 (MMAT; Hong et al., 2018; two independent raters, $\kappa = 0.82$)	Yes
12	Specify the effect measure(s) used in the synthesis or presentation of results.	Not applicable — narrative/thematic synthesis used instead of meta-analysis; rationale given in Section 3.4	N/A

Item #	Checklist item	Location in manuscript	Reported
13a	Describe the process used to decide which studies were eligible for each synthesis.	Section 3.4	Yes
13b	Describe methods for preparing data for presentation or synthesis.	Section 3.4 (extraction matrix; Table 3)	Yes
13c	Describe methods for tabulating or visually displaying results.	Tables 3–4; Figures 3–4	Yes
13d	Describe methods used to synthesize results.	Section 3.4 (three-stage thematic synthesis; Thomas and Harden, 2008)	Yes
13e	Describe methods for exploring possible causes of heterogeneity among study results.	Section 3.4 (thematic synthesis and cross-study comparison of study characteristics, AI functions, and SRL phases)	Yes
13f	Describe sensitivity analyses to assess robustness of synthesized results.	Section 4.3.2 — a qualitative sensitivity consideration was conducted by examining the influence of a lower-quality study on interpretation of findings.	Partial
14	Describe methods for assessing risk of bias from missing results (e.g., publication bias).	Section 5.5 — acknowledged as a limitation (grey literature not searched); not a formal pre-specified assessment	Partial
15	Describe methods for assessing certainty (confidence) in the body of evidence for each outcome.	Section 5 (Discussion opening) — evidence confidence interpreted considering MMAT appraisal, study design limitations, and consistency of findings	Partial
**RESULTS**			
16a	Report numbers of records screened, assessed for eligibility, and included, with reasons for exclusion at each stage.	Figure 2 (PRISMA flow diagram); Section 3.3	Yes
16b	Cite studies that appeared eligible but were excluded, and explain why.	Section 3.3 (three excluded studies characterized by reason)	Yes
17	Cite each included study and present its characteristics.	Table 3 (descriptive characteristics and data extraction matrix)	Yes
18	Present risk-of-bias assessments for each included study.	Table 2 (MMAT appraisal, per criterion)	Yes
19	Present results for all outcomes, for each study.	Section 4.3; Table 3 (Key Findings column)	Yes
20a	Briefly summarize the characteristics and risk of bias of studies contributing to each synthesis.	Sections 4.1 and 3.5	Yes
20b	Present results of all syntheses conducted.	Sections 4.2–4.3; Table 4 (SRL-phase × tool-category mapping)	Yes
20c	Present results of investigations of heterogeneity among study results.	Sections 4.1 and 5.5 — methodological and contextual heterogeneity summarized narratively	Partial
20d	Present results of any sensitivity analyses.	Section 4.3.2 (Xu and Fan, 2025 sensitivity note)	Partial
21	Present an assessment of risk of bias from missing results for each synthesis.	Section 5.5 (publication/positive-result bias discussed as a limitation)	Partial
22	Present an assessment of certainty/confidence in the body of evidence for each outcome.	Section 5 (Discussion opening paragraph)	Partial

Item #	Checklist item	Location in manuscript	Reported
**DISCUSSION**			
23a	Provide a general interpretation of results in the context of other evidence.	Sections 5.1–5.4	Yes
23b	Discuss limitations of the evidence included in the review.	Sections 3.5 and 5.5	Yes
23c	Discuss limitations of the review processes used.	Section 5.5 (English-only corpus, no protocol registration, no grey-literature search, self-report SRL measures)	Yes
23d	Discuss implications of the results for practice, policy, and future research.	Sections 5.4 and 5.5 (Future Research Priorities: methodological / theoretical / practical clusters)	Yes
**OTHER INFORMATION**			
24a	Provide registration information, including register name and number, or state that the review was not registered.	Section 5.5 — states the review was not prospectively registered on PROSPERO or OSF	Yes
24b	Indicate where the review protocol can be accessed, or state that no protocol was prepared.	Sections 3.5	Yes
24c	Describe and explain any amendments to information provided at registration or in the protocol.	Not applicable — no protocol was registered	N/A
25	Describe sources of financial or non-financial support for the review, and the role of funders.	Funding Information / Funding Statement	Yes
26	Declare any competing interests of review authors.	Conflict of Interest Statement	Yes
27	Report which of the following are publicly available and where: template data collection forms, extracted data, analytic code, and other materials used in the review.	The data extraction form and coding framework used in this review are available from the corresponding author upon reasonable request	Yes
